# Glucose-Assisted Synthesis of Porous, Urchin-like Co_3_O_4_ Hierarchical Structures for Low-Concentration Hydrogen Sensing Materials

**DOI:** 10.3390/ma17061364

**Published:** 2024-03-16

**Authors:** Xin Deng, Xiao Zhang, Xiaochuan Long, Xiaopeng Liu

**Affiliations:** 1 State Key Laboratory of Advanced Materials for Smart Sensing, China GRINM Group Co., Ltd., Beijing 100088, China; 2 GRIMAT Engineering Institute Co., Ltd., Beijing 101407, China; 3 General Research Institute for Nonferrous Metals, Beijing 100088, China

**Keywords:** Co_3_O_4_, urchin-like, glucose, hydrogen, gas sensing

## Abstract

The Co_3_O_4_ is a typical p-type metal oxide semiconductor (MOS) that attracted great attention for hydrogen detection. In this work, porous, urchin-like Co_3_O_4_ was synthesized using a hydrothermal method with the assistance of glucose and a subsequent calcination process. Urchin-like Co_3_O_4_ has a large specific surface area of 81.4 m^2^/g. The response value of urchin-like Co_3_O_4_ to 200 ppm hydrogen at 200 °C is 36.5 (R_g_/R_a_), while the low-detection limit is as low as 100 ppb. The obtained Co_3_O_4_ also exhibited good reproducibility, long-term stability, and selectivity towards various gases (e.g., ammonia, hydrogen, carbon monoxide, and methane). Porous, urchin-like Co_3_O_4_ is expected to become a potential candidate for low-concentration hydrogen-sensing materials with the above advantages.

## 1. Introduction

Hydrogen, a futuristic and ideal clean energy source, is widely used in the fields of biomedicine, metal smelting, chemical production, and fuel cells [1,2,3]. However, the properties of hydrogen, such as its wide range of explosion concentration (4.0–74.5% in the air), low ignition energy (0.018 MJ), and high explosion index (550, 10 times of methane), limit its application [4,5]. Hydrogen sensing is an efficient way to monitor hydrogen concentration and promote hydrogen applications. Therefore, the need to develop and apply practical sensing materials to high-performance hydrogen sensors is urgent.

Co_3_O_4_, as a p-type MOS with a spinel structure, is a promising gas-sensing material due to its high stability, good humidity resistance, and high catalytic activity [6,7]. However, Co_3_O_4_ still suffers from having a high detection limit, low response, and poor selectivity. The regulation of morphology is an effective way to improve Co_3_O_4_ sensing performance, such as in nanorods [8], nanowires [9], nanodisks [10], nanotubes [11], nanoflowers [12,13], etc. For example, Qiu et al. synthesized needle-like Co_3_O_4_ for ethanol sensing using a hydrothermal method that has a response value of 19.6 to 100 ppm ethanol at 160 °C [14]. Fang et al. fabricated MOF-derived Co_3_O_4_ hollow nanotubes, which have a detective limit of 10 ppm to toluene [15]. Three Co_3_O_4_ samples with different morphologies were synthesized by Zhang et al. to investigate structure-dependent gas-sensing properties. The results showed that the sensing performance of rod-assembled spheres of Co_3_O_4_ and sheet-assembled flowers of toluene outperformed cube-shaped Co_3_O_4_ [16]. One-dimensional nanostructures show good sensing performance due to their higher specific surface area, electron transfer efficiency, and surface energy; also, the material size reaches the Debye length [17]. However, studies of one-dimensional Co_3_O_4_ materials conducted for hydrogen are rare. Therefore, it is necessary to investigate further one-dimensional Co_3_O_4_ with a highly specific surface area and porosity for high-performance hydrogen-sensing materials.

In this work, porous, urchin-like Co_3_O_4_ was synthesized through a hydrothermal process, with the assistance of glucose, followed by a calcination treatment. The hierarchical structure retained the advantage of a one-dimensional nanoneedle. Meanwhile, it also avoids the agglomeration of conventional nanoneedle materials and provides more active sites for gas adsorption. Thus, the gas diffusion channels were well preserved. The influence of the additive glucose dosage on the morphology of Co_3_O_4_ was investigated, and a possible formation mechanism of urchin-like hierarchical structure was elucidated. The hydrogen-sensing performance of urchin-like Co_3_O_4_ was tested, while the structure was also characterized using SEM, TEM, XRD, and XPS. The obtained results were beneficial for clarifying the sensing mechanism of Co_3_O_4_ and, thus, contributed to expanding hydrogen-sensing materials. 

## 2. Experimental Section

### 2.1. Preparation of Porous, Urchin-like Co_3_O_4_ Hierarchical Structure

All chemicals were purchased from Shanghai Aladdin Reagent Co., Ltd., Shanghai, China. All chemical reagents were used directly and did not require secondary purification.

A total of 0.146 g Co(NO_3_)_2_·6H_2_O, 0.180 g urea, and a fixed mass of glucose (the mass ratio of glucose/Co(NO_3_)_2_·6H_2_O was 0, 0.25, 0.5, and 0.75, respectively) were added to a mixed solvent (10 mL of ethanol and 20 mL of deionized water). After magnetic stirring for 1 h, the mixed solution was transferred to a 50 mL Teflon-sealed autoclave and kept in an oven at 110 °C for 12 h. Subsequently, after being air-cooled to room temperature, a pinkish–purple precipitate (Co_3_O_4_ precursors) was obtained by washing several times with deionized water and ethanol and dried at 60 °C for 12 h. According to the TGA curves of the precursors (Figure S2), the calcination treatment was set to 400 °C for 2 h to obtain mixed-valent Co_3_O_4_ and protect the urchin-like structure from collapse. The obtained precursors are named G_x_Co_3_O_4_, where x represents the mass ratio of glucose to cobalt nitrate hexahydrate: x = 0, 0.25, 0.5, and 0.75. It is accordingly named T_400_G_x_Co_3_O_4_ after calcination at 400 °C.

### 2.2. Characterization Methods

The morphology and structure of the samples were characterized using scanning electron microscopy (SEM, JEOL JSM-7900F, Tokyo, Japan) and transmission electron microscopy (TEM, JEOL2100 PLUS, Tokyo, Japan). Thermogravimetric analyzer analysis (TGA, Mettler Toleto TGA-DSC3+, Zurich, Switzerland) was conducted in an air atmosphere at a heating rate of 10 °C/min between 25 °C and 1000 °C. The crystal-phase composition and structure of the samples were characterized using X-ray diffraction (XRD, Smartlab KD2590N, Rigaku, Tokyo, Japan) with Cu Kα source (λ = 0.15418). X-ray photoelectron spectroscopy (XPS, ESCALAB Xi+, Al Kα source, 1486.6 eV, Thermo Fisher Scientific, Waltham, MA, USA) was used to analyze the elemental composition and chemical state of the samples. The specific surface area of the sample was calculated using Brunauer–Emmett–Teller (BET, ASAP 3020, Micromeritics, Norcross, GA, USA), and the pore size information of the sample was calculated using the Barrett–Joyner–Helenda (BJH) method with N_2_ isothermal adsorption–desorption testing at 77 K. 

### 2.3. Measurement of Gas-Sensing Performance

A gas-sensing system (JF02, Gui Yan Jin Feng Technology Co., Ltd., Guizhou, China) was used to conduct gas-sensing testing of the materials. A simple schematic diagram of the JF02 gas-sensing system is shown in Figure S1. All gases used during testing were injected via external gas cylinders and mixed in the “Mixture Chamber”. The mass flow and concentration of gases were controlled by the “Mass Flow” module, which was monitored through a flowmeter. The temperature control in the sensing process was achieved through the heating platform in the enclosed “Test Chamber”. The changes in resistance signal were captured by a pair of electrode probes in the “Test Chamber”, processed by the “Test Module”, and transmitted into a PC. In a typical test process, the as-prepared Co_3_O_4_ samples (about 50 mg) were ultrasonically dispersed in ethanol (mass ratio 1:20) for 10 min to obtain a homogeneous suspension. Subsequently, 10 μL of the suspension was dropped onto an alumina-ceramic sheet equipped with Ag electrodes using a pipette and then dried. Finally, the electrode sheet covered with a layer of the sample was placed in the test chamber of the gas-sensing system and connected to the electrodes in the chamber. The chamber was heated by a heating platform, which was surrounded by a pair of probes. The resistance of each sample was measured by the probes during testing. All gas-sensing measurements were conducted in a closed gas chamber, with a response (R) defined as R = R_g_/R_a_, where R_g_ and R_a_ are the resistance of the sample in the target gas and air, respectively. Additionally, the response time (τ_res_) is defined as the time required for the sample resistance to reach 90% of its maximum value, and the recovery time (τ_rec_) is defined as the time required for the sample resistance to decrease to 10% of the overall resistance change. The selective coefficient is an evaluation of the selectivity of a gas sensor. It is defined as the ratio of the gas sensor’s sensitivity between the target gas and other interfering gases. For a hydrogen sensor, it is calculated as S_c_ = S_hydrogen_/S_interfering gases_.

## 3. Results and Discussion

### 3.1. Morphology and Structure of Porous, Urchin-like Co_3_O_4_

**Figure 1:**
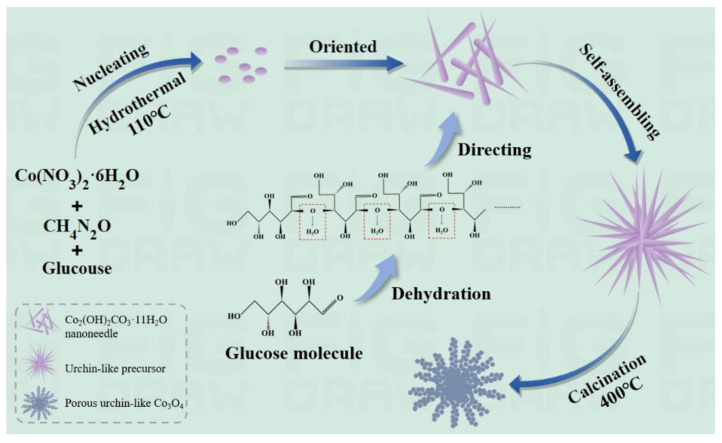
Schematic illustrations of the growth mechanism of porous, urchin-like Co_3_O_4_.

In this work, glucose played a key role in the morphology regulation of the precursor, acting as the structural directing agent. Figure 1 illustrates the formation process of porous, urchin-like Co_3_O_4_. During the hydrothermal process, urea continuously hydrolyzed and released OH^−^ and ${\mathrm{CO}}_{3}^{2-}$ ions, which then reacted with Co^2+^ ions provided by Co(NO_3_)_2_·6H_2_O in solution. This reaction formed crystal nuclei that grew longitudinally along the polysaccharide molecular chains resulting from the dehydration of glucose molecules, forming needle-like structures [18]. Subsequently, the nanoneedles self-assemble to form the Co_3_O_4_ precursor, as indicated by the XRD pattern (Figure S3), to be Co_2_(OH)_2_CO_3_·11H_2_O after oven drying. The porous Co_3_O_4_ phase formed after Co_2_(OH)_2_CO_3_·11H_2_O releases CO_2_ and H_2_O gas during calcination treatment in an air atmosphere. The reactions that occurred in the hydrothermal system can be presented as follows:

(1) $${\mathrm{NH}}_{2}{\mathrm{CONH}}_{2}{+3H}_{2}O\overset{\mathrm{Hydrolysis}}{\to }{2\mathrm{NH}}_{4}^{+}{+\mathrm{HCO}}_{3}^{-}{+\mathrm{OH}}^{-}$$

(2) $${\mathrm{HCO}}_{3}^{-}\to {\mathrm{CO}}_{3}^{2-}{+H}^{+}$$

(3) $${2\mathrm{Co}}^{2+}{+2\mathrm{OH}}^{-}{+\mathrm{CO}}_{3}^{2-}\to {\mathrm{Co}}_{2}{\left(\mathrm{OH}\right)}_{2}{\mathrm{CO}}_{3}$$

(4) $${\mathrm{Co}}_{2}{\left(\mathrm{OH}\right)}_{2}{\mathrm{CO}}_{3}{+11H}_{2}O\overset{\mathrm{Dry}}{\to }{\mathrm{Co}}_{2}{\left(\mathrm{OH}\right)}_{2}{\mathrm{CO}}_{3}\cdot {11H}_{2}O$$

(5) $${3\mathrm{Co}}_{2}{\left(\mathrm{OH}\right)}_{2}{\mathrm{CO}}_{3}\cdot {11H}_{2}{O+O}_{2}\overset{400\;{}^{\circ}C}{\to }{2\mathrm{Co}}_{3}O_{4}{+3\mathrm{CO}}_{2}{+14H}_{2}O$$

**Figure 2:**
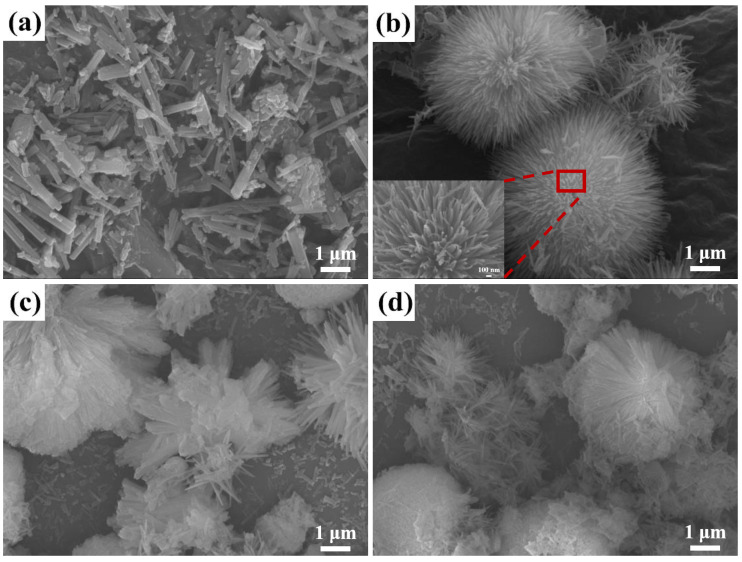
SEM images of Co_3_O_4_ precursors synthesized with different glucose amounts: (**a**) G_0_Co_3_O_4_; (**b**) G_0.25_Co_3_O_4_; (**c**) G_0.5_Co_3_O_4_; (**d**) G_0.75_Co_3_O_4_.

The SEM images of the Co_3_O_4_ precursor are shown in Figure 2. The precursor exhibits a nanorod-shaped structure with no glucose (x = 0) in the fabrication process (Figure 2a). When the glucose amount is kept at x = 0.25, an ordered urchin-like Co_3_O_4_ precursor (Figure 2b) consisting of basic nanoneedle structures is formed under the guidance of the glucose molecular chain. From the high-magnification SEM images of the urchin-like structure precursor shown in the inset of Figure 2b, the ordered nanoneedle arrays can be seen, which is beneficial for gas molecule adsorption in the sensing process. With the increase in the amount of glucose (x = 0.5), the density of the urchin-like Co_3_O_4_ increases due to the agglomeration of nanoneedles (Figure 2c). The spines of the “urchin” transform from nanoneedle to rough rod-shaped structures. When the glucose amount further increases to x = 0.75, the urchin-like Co_3_O_4_ exhibits a nearly block-like structure (Figure 2d), which is not conducive to obtaining a high specific surface area. The main reason for agglomeration is the curling, aggregation, and branching of polysaccharide molecular chains, reducing the linear growth space of transient crystal nuclei. The results show that the optimal mass of glucose to fabricate the urchin-like Co_3_O_4_ precursor is 0.037 g (x = 0.25).

**Figure 3:**
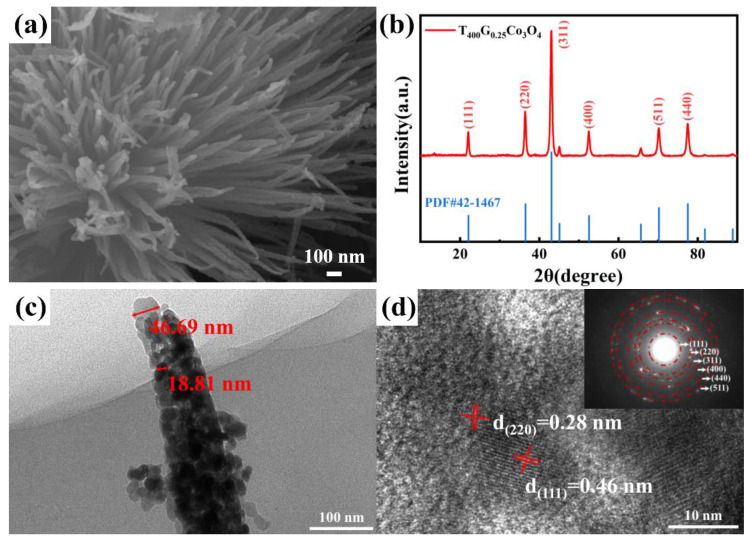
Porous, urchin-like T_400_G_0.25_Co_3_O_4_: (**a**) SEM image; (**b**) XRD pattern; (**c**) TEM image; (**d**) HRTEM image and SAED image (inset).

Figure 3a shows the SEM images of porous, urchin-like T_400_G_0.25_Co_3_O_4_, which was calcinated at 400 °C for 2 h. The porous structure forms because the OH^−^ and ${\mathrm{CO}}_{3}^{2-}$ ions and residual glucose molecules are converted into H_2_O and CO_2_ gas molecules during the calcination process and then escape [19]. Ultimately, a porous, urchin-like Co_3_O_4_ hierarchical structure is self-assembled with nanoneedles, and the nanoneedles are shaped from nanoparticles. Other T_400_G_x_Co_3_O_4_ samples are shown in Figure S4. After the calcinating process, rod-shaped G_0_Co_3_O_4_ seriously aggregates into blocks with a diameter of about 1 μm, and T_400_G_0_Co_3_O_4_ is tightly composed of nanoparticles with a few stacked pores (Figure S4a). A partial thick nanoneedle structure (about 160 nm) is retained in T_400_G_0.5_Co_3_O_4_ (Figure S4b). It exhibits a disorderly stacked structure, which reduces the gas adsorption and dissociation area in the gas-sensing process. T_400_G_0.75_Co_3_O_4_ illustrates the structure of a near-solid microsphere with a diameter of about 18 μm (Figure S4c). The structure of T_400_G_0.25_Co_3_O_4_ tends to obtain a high specific surface area, but others are contrary to that.

Figure 3b shows the XRD pattern of T_400_G_0.25_Co_3_O_4_ with sharp diffraction peaks, indicating high crystallinity. The characteristic peaks at 2θ = 22.1, 36.4, 43.1, 52.5, 70.2, and 77.5° correspond to the (111), (220), (311), (400), (511), and (440) crystal planes of the spinel Co_3_O_4_ (PDF#45-1467), respectively. The Co_2_(OH)_2_CO_3_·11H_2_O has completely transformed into a spinel Co_3_O_4_ single-phase after the calcination process at 400 °C for 2 h. 

The morphology and crystal structure of the obtained porous, urchin-like Co_3_O_4_ were further tested by TEM. The basic nanoneedle structure is composed of nanoparticles (Figure 3c), and the nanoneedle diameter of T_400_G_0.25_Co_3_O_4_ is about 60 nm, which is smaller than others. The smaller size of nanomaterials provides advantages for gas-sensing, especially the 1D nanomaterials that have reached Debye length [18]. From the HRTEM image (Figure 3d), lattice fringes of 0.28 nm and 0.46 nm correspond to the (220) and (111) crystal planes of spinel Co_3_O_4_ after Fourier transform. The lack of clarity in the lattice fringes of 0.28 nm is probably due to the thickness of the sample. Research has shown that exposure of (111) crystal planes of Co_3_O_4_ is beneficial for oxygen adsorption, leading to a wider hole accumulation layer (HAL) [20]. The concentric rings presented in the SAED (inset of Figure 3d) indicate the polycrystalline structure of porous, urchin-like Co_3_O_4_.

**Figure 4:**
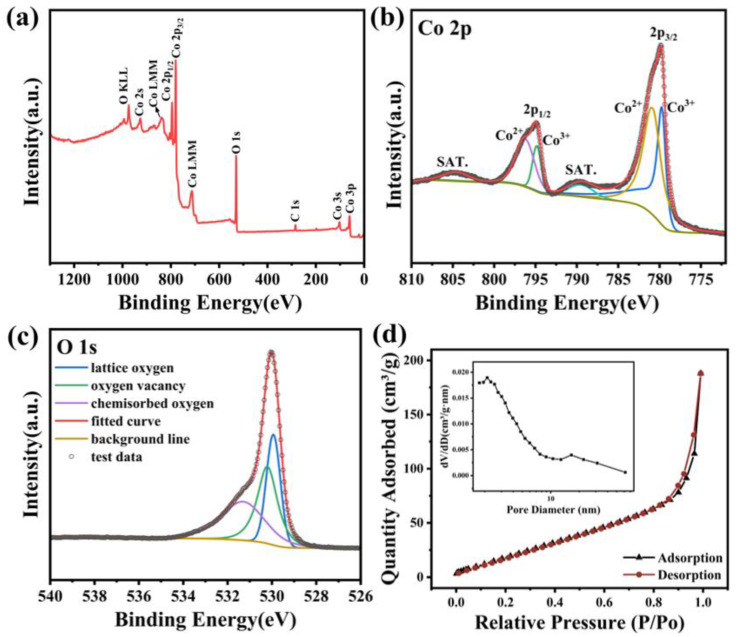
T_400_G_0.25_Co_3_O_4_: (**a**) XPS survey spectrum; (**b**) High-resolution XPS spectrum of Co 2p; (**c**) High-resolution XPS spectrum of O 1s; (**d**) Nitrogen adsorption–desorption isotherms and BJH pore size distribution curve (insert).

The XPS spectra are shown in Figure 4a–c, which was conducted to further clarify the elemental composition and valence state of urchin-like Co_3_O_4_. According to the XPS survey spectrum (Figure 4a), the peaks located at 284.8, 530.8, 780.8, and 795.8 eV correspond to C 1s, O 1s, Co 2p_3/2_, and Co 2p_1/2_, respectively. The Co 2p_3/2_ peak can be divided into two peaks with binding energies of 779.8 eV and 781.5 eV, representing Co^3+^ 2p_3/2_ and Co^2+^ 2p_3/2_ (Figure 4b). The Co 2p_1/2_ can be divided into two peaks at 794.8 eV and 797.6 eV, representing Co^3+^ 2p_1/2_ and Co^2+^ 2p_1/2_ [21]. The results certify the coexistence of Co^2+^ and Co^3+^ chemical states on the material surface. The asymmetric O 1s peak (Figure 4c) can be fitted and differentiated into three peaks, corresponding to lattice oxygen (529.8 eV), oxygen vacancies (530.2 eV), and chemically adsorbed oxygen (531.3 eV) [22]. The proportion of oxygen vacancies was calculated to be 24.7%, which is beneficial for oxygen adsorption and dissociation on the material surface [23].

N_2_ adsorption–desorption isotherms were tested to investigate the specific surface area and pore structure of the obtained Co_3_O_4_. The results are summarized in Table 1. The porous, urchin-like T_400_G_0.25_Co_3_O_4_ has the largest specific surface area of 81.4 m^2^/g, approximately twice that of the rod-shaped T_400_G_0_Co_3_O_4_. The specific surface area of the obtained material was inversely proportional to the glucose content (when x = 0.25, 0.5, and 0.75). It displayed a typical type IV adsorption isotherm with an H3-type hysteresis loop (Figure 4d), indicating a mesoporous structure [24]. T_400_G_0.25_Co_3_O_4_ has the smallest pore size and highest porosity, which benefits the diffusion and dissociation of H_2_ (small molecule) on the material surface, improving the transmission efficiency of the H atom [25].

### 3.2. Hydrogen-Sensing Performance of Porous, Urchin-like Co_3_O_4_

**Figure 5:**
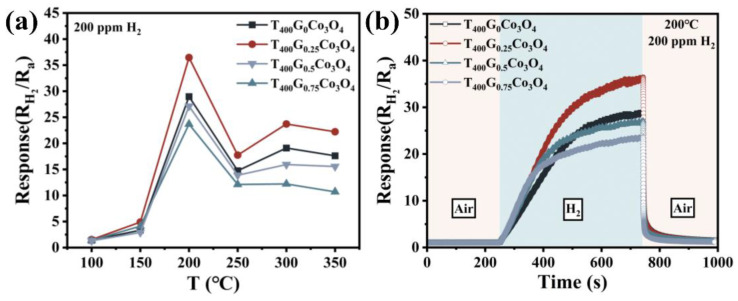
T_400_G_x_Co_3_O_4_ material: (**a**) Response to 200 ppm H_2_ at different operating temperatures; (**b**) The dynamic response–recovery curve for 200 ppm H_2_ at 200 °C.

The operating temperature significantly impacts gas-sensing response due to the kinetics and mechanics of gas adsorption and desorption [21,26]. Figure 5a shows the response values of T_400_G_x_Co_3_O_4_ to 200 ppm H_2_ over a temperature range of 100–350 °C. With increasing operating temperature, the response values of T_400_G_x_Co_3_O_4_ (x = 0, 0.25, 0.5, and 0.75) initially increase and then decrease. The optimal operating temperature is found to be 200 °C. This can be attributed to weak reaction kinetics between H_2_ and T_400_G_x_Co_3_O_4_ at lower temperatures, while desorption of H_2_ on the T_400_G_x_Co_3_O_4_ surface dominates at higher temperatures, leading to a reduction in the response value. Furthermore, T_400_G_0.25_Co_3_O_4_ exhibits the highest response value (R_g/_R_a_ = 36.5), which decreases with increasing glucose content. This is attributed to the largest specific surface area and the smallest nanoneedle diameter of T_400_G_0.25_Co_3_O_4_, providing more active sites for gas adsorption and dissociation, facilitating easier reaction between H_2_ and the oxygen anion on the material surface. The dynamic response–recovery curve at 200 °C (Figure 5b) shows a fast recovery time of 9.1 s. However, the porous structure and multi-layer adsorption mechanism, as presented by the type IV nitrogen adsorption–desorption isotherms curve (Figure 4d) of the material, results in a relatively long response time (178 s). This occurs because H_2_ molecules take more time to reach the surface of the inner layer of the material for adsorption and reaction. The above indicates that the porous, urchin-like Co_3_O_4_ is much more sensitive to H_2_. Based on the above, subsequent gas-sensing performance tests, such as response to different H_2_ concentrations, selectivity, and repeatability, have been conducted at the optimal operating temperature (200 °C).

**Figure 6:**
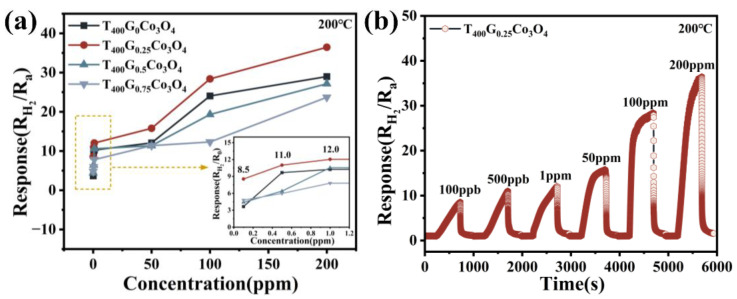
(**a**) Response to different concentrations of H_2_ at 200 °C; (**b**) Dynamic response–recovery curve of T_400_G_0.25_Co_3_O_4_ to different concentrations of H_2_ at 200 °C.

The response curves of T_400_G_x_Co_3_O_4_ materials towards 0.1–200 ppm H_2_ at 200 °C were further investigated (Figure 6). The insufficient response to low-concentration hydrogen remains one of the urgent challenges for current MOS hydrogen-sensing materials. Figure 6a shows that T_400_G_0.25_Co_3_O_4_ exhibits a higher response than others at various H_2_ concentrations, indicating the optimal ratio of glucose is m_G_/m_Co_ = 0.25. Moreover, the dynamic response curve of T_400_G_0.25_Co_3_O_4_ (Figure 6b) shows a high response value (8.5) at extremely low H_2_ concentrations (100 ppb) and returns to the initial state after introducing air. The unique nanoneedle arrays of the “urchin” structure provide ordered pore channels for H_2_ molecule transmission, resulting in sufficient gas response even at low H_2_ concentrations.

**Figure 7:**
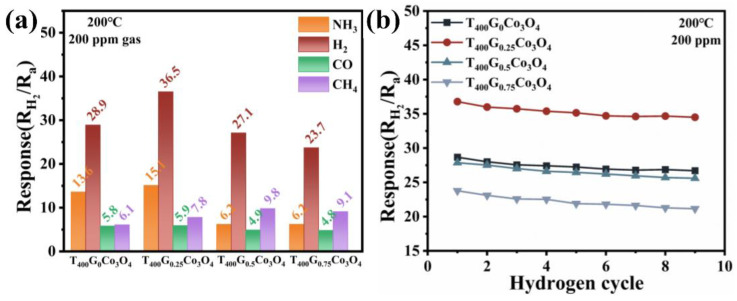
T_400_G_x_Co_3_O_4_ material at 200 °C: (**a**) Response values to different reducing gases; (**b**) Response value after 9 hydrogen cycles.

The responses of T_400_G_x_Co_3_O_4_ to various reducing gases (ammonia, hydrogen, carbon monoxide, and methane) at 200 °C were tested to further investigate the selectivity of T_400_G_x_Co_3_O_4_. The results are shown in Figure 7a. The selectivity coefficients of T_400_G_0.25_Co_3_O_4_ to NH_3_, CO, and CH_4_ have been calculated to be 2.42, 6.19, and 4.68, respectively. Others are also shown in Table 2. A larger selectivity coefficient indicates better selectivity. It is generally considered to have excellent selectivity when the selectivity coefficient of the gas sensor is greater than 3. It exhibits more excellent sensitivity to H_2_ than other counterparts at the same concentration, mainly due to the porous structure of the material acting as a molecular sieve for gas molecules with different sizes. The narrow pore channel allows small size molecules of H_2_ to transit and collide with the material surface, leading to a stronger gas reaction. Repeatability refers to the degree to which the sensor deviates from the measurement result after repeated use in the case of a certain gas concentration. The response values and dynamic curves of T_400_G_x_Co_3_O_4_ to 200 ppm H_2_ at 200 °C after 9 reversible cycles are shown in Figure 7b and Figure S6. The response value of the materials only presents a decrease of 1.9–2.6 after 9 reversible cycles, indicating an excellent repeatability of the materials.

### 3.3. Hydrogen-Sensing Mechanism and Performance Analysis of Porous, Urchin-like Co_3_O_4_

#### 3.3.1. Hydrogen-Sensing Mechanism of Co_3_O_4_

**Figure 8:**
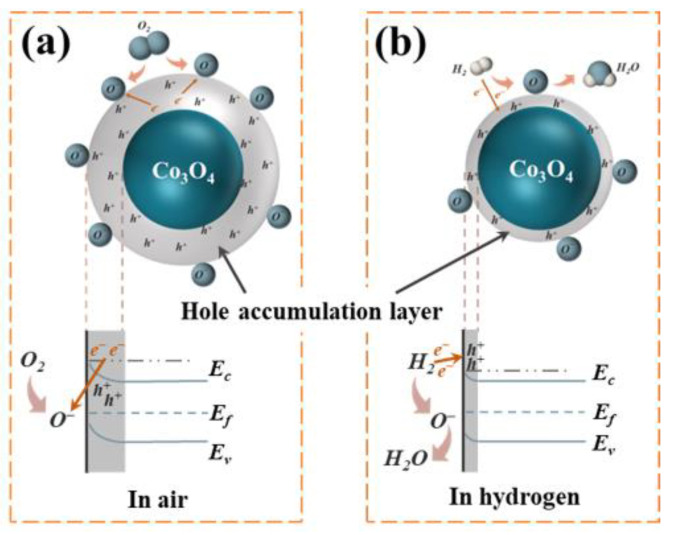
Schematic diagram of gas-sensing mechanism and energy-band changes in Co_3_O_4_ material: (**a**) in air; (**b**) in hydrogen.

The sensing mechanism of MOS-type hydrogen-sensing materials is based on the resistance variation mediated by surface chemistry [27,28,29]. Therefore, the specific surface area is one critical factor affecting the gas-sensing properties [30]. As a p-type MOS, the main charge carrier of Co_3_O_4_ is holes. When Co_3_O_4_ is exposed to air (Figure 8a), O_2_ absorbs on the surface of the material and then dissociates into oxygen anions ($O_{2}^{-}$, O^−^, O^2−^) by seizing electrons from the conduction band of the material. Different oxygen anions are formed at different temperatures, as shown in Equations (6)–(9) since chemisorption is an energy-activated process [31]. As shown in Figure 8a, O_2_ dissociates to O^−^ at 200 °C (the optimal operation temperature in this work) on the Co_3_O_4_ surface. Meanwhile, the energy band bends upwards, forming a hole accumulation layer (HAL), which leads to a decrease in resistance [32]. When Co_3_O_4_ is exposed to H_2_ (Figure 8b), the subsequent hydrogen-sensing process occurs through a chemical reaction between O^−^ and H_2_ (Equation (10)). This process releases the electrons back to the HAL and narrows them, increasing material resistance.

O_2(g)_ → O_2(ads)_ (6)

O_2(ads)_ + e^−^ → O_2_^−^_(ads)_ (T < 150 °C) (7)

O_2_^−^_(ads)_ + e^−^ → 2O^−^_(ads)_ (150 °C < T < 400 °C) (8)

O^−^_(ads)_ + e^−^ → O^2−^_(ads)_ (T > 400 °C) (9)

H_2_ + O^−^_(ads)_ → H_2_O_(gas)_ + e^−^ (10)

#### 3.3.2. Analysis of Hydrogen-Sensing Performance of Porous, Urchin-like Co_3_O_4_

The response value and detection limit of the obtained material to H_2_ are comparable to other competing sensing materials in Table 3. MOS gas-sensing materials of different structures were listed in the table and compared regarding the optimal working temperature, response at a certain concentration, and detection limit to this work. To ensure the comparability of data from references, the different response calculation methods of these references have been unified and provided as annotations under the table. The sensing material obtained in this work shows great advantages in terms of sensitivity and detection limits that have reached the ppb level. Considering the hydrogen-sensing mechanism of Co_3_O_4_, the performance of the porous, urchin-like Co_3_O_4_ was enhanced by excellent structure regulation. According to the results of the structure analysis, the T_400_G_0.25_Co_3_O_4_ material had an ultra-high specific surface area of 81.4 m^2^/g and a high porosity of 0.449 cm^3^/g. It provided sufficient active sites for adsorption and dissociation of H_2_ on the material surface, which, combined with high O^−^ coverage, further promoted the redox reaction during the sensing process [33].

Moreover, the T_400_G_0.25_Co_3_O_4_ had the optimal pore size of 11 nm, which provides an effective diffusion channel for H_2_. At this pore size, it was conducive to the diffusion of small hydrogen molecules and played a screening role in large molecule gases [40]. The diffusion of gas molecules in pores is related to the relationship between the mean free path and pore size of gas molecules, which is derived from three types of diffusion process: volume diffusion, Knudsen diffusion, and transition diffusion [41]. The diffusion way of H_2_ in the T_400_G_0.25_Co_3_O_4_, which has an 11 nm porous size, belongs to Knudsen diffusion. In this way, the H_2_ molecule can smoothly pass through the pore channel and collide with the material surface, promoting the adsorption and dissociation of H_2_ on the T_400_G_0.25_Co_3_O_4_ surface. Meanwhile, large molecule gas tends to undergo transition diffusion and volume diffusion. Therefore, the porous structure of T_400_G_0.25_Co_3_O_4_ resulted in high sensitivity at low hydrogen concentrations and further increased the hydrogen selectivity of the material.

In addition, the high-resolution spectrum of Co 2p in the XPS (Figure 4b) illustrated the coexisting chemical states of Co^2+^ and Co^3+^ of the material. The conversion of two types of ions can create additional active sites and induce redox reactions, which improve the sensitivity of the material to different concentrations of H_2_ [42,43,44].

## 4. Conclusions

In summary, porous, urchin-like Co_3_O_4_ was controllably synthesized through a hydrothermal method followed by a calcination process for hydrogen detection. The Co_3_O_4_ exhibited excellent hydrogen detection performance, with a response value (8.5) to 100 ppb hydrogen that is superior to other morphology Co_3_O_4_ samples. The ratio of glucose to cobalt salt greatly affected the structure of Co_3_O_4_, and porous, urchin-like T_400_G_0.25_Co_3_O_4_ material can be obtained while the m_G_/m_Co_ ratio is 0.25. It exhibits optimum hydrogen-sensing performance, which can be attributed to (1) the ultra-large specific surface area, providing more active sites, (2) the high porosity and appropriate pore size corresponding to Knudsen diffusion, and (3) the conversion between Co^2+^ and Co^3+^ of Co_3_O_4_, which enhances catalytic activity and promotes sensing performance.

## Figures and Tables

**Table 1:** Specific surface area and pore size of T_400_G_x_Co_3_O_4_.

Sample	Specific Surface Areas (m^2^/g)	Average Pore Size (nm)	Pore Volume (cm^3^/g)
T_400_G_0_Co_3_O_4_	42.7	32.7	0.285
T_400_G_0.25_Co_3_O_4_	81.4	11.0	0.449
T_400_G_0.5_Co_3_O_4_	57.5	20.3	0.379
T_400_G_0.75_Co_3_O_4_	55.3	22.6	0.407

**Table 2:** The selectivity coefficients of T_400_G_x_Co_3_O_4_.

Sample	NH_3_	CO	CH_4_
T_400_G_0_Co_3_O_4_	2.13	4.98	4.74
T_400_G_0.25_Co_3_O_4_	2.42	6.19	4.68
T_400_G_0.5_Co_3_O_4_	4.37	5.53	2.77
T_400_G_0.75_Co_3_O_4_	3.82	4.94	2.60

**Table 3:** Comparison of MOS-sensing materials for H_2_.

Materials	Structure	Working Temperature (°C)	Concentration (ppm)	Response	Detection Limit (ppm)	Ref.
Pd-SnO_2_/Co_3_O_4_	nanoparticle	300	100	57.9	10	[34]
NiO	nanofilm	250	200	35.7 *	50	[35]
1 at.% Pt-ZnO	pencil-like microrods	150	100	2.8 *	10	[27]
Pt-Fe_2_(MoO_4_)_3_	nanoflower	300	10	3.1 *	1	[36]
In_2_O_3_	octahedra	260	500	14	4	[37]
V_2_O_5_	hollow structure	25	200	2.9	10	[38]
MnCo_2_O_4_/r-GO	flower	160	250	1.1	100	[39]
T_400_G_0.25_Co_3_O_4_	urchin-like	200	200	36.5	0.1	This work

* All the response values calculated by $S\%=\frac{{|R}_{a}{\;-\;R}_{g}|}{R_{g}}\times \%$ were converted to R_a_/R_g_ (n-type MOS) or R_g_/R_a_ (p-type MOS).

## Data Availability

Data are contained within the article.

## Supplementary Materials

The following supporting information can be downloaded at https://www.mdpi.com/article/10.3390/ma17061364/s1, Figure S1: Schematic diagram of JF02 system; Figure S2: TGA curves of Co_3_O_4_ precursors; Figure S3: XRD pattern of G_0.25_Co_3_O_4_; Figure S4: SEM images of T_400_G_x_Co_3_O_4_: (a) T_400_G_0_Co_3_O_4_; (b) T_400_G_0.5_Co_3_O_4_; (c) T_400_G_0.75_Co_3_O_4_; Figure S5: Response to different concentrations of H_2_ at 200 °C: (a) T_400_G_0_Co_3_O_4_; (b) T_400_G_0.25_Co_3_O_4_; (c) T_400_G_0.5_Co_3_O_4_; (d) T_400_G_0.75_Co_3_O_4_; Figure S6: Dynamic response curve of T_400_G_x_Co_3_O_4_ at 200 °C after 9 hydrogen cycles.
