# Supplementary material for: Glucose-Assisted Synthesis of Porous, Urchin-like Co3O4 Hierarchical Structures for Low-Concentration Hydrogen Sensing Materials
Source: Materials (Basel). 2024 Mar 16;17(6):1364. doi: 10.3390/ma17061364 (PMC10972426; doi:10.3390/ma17061364)
Supplement: Supplementary file 1 [file materials-17-01364-s001.zip › materials-2893740-supplementary.pdf]

Supporting information

Glucose-assisted synthesis of porous urchin-like Co_3O_4 hierarchical structures for low-concentration hydrogen sensing materials

Xin Deng[a,b,c], Xiao Zhang[a,b,*], Xiaochuan Long[a,b,c], Xiaopeng Liu[a,b,c,*]

[a]*State Key Laboratory of Advanced Materials for Smart Sensing, China GRINM Group Co., Ltd., Beijing 100088, China*

[b]*GRIMAT Engineering Institute Co., Ltd., Beijing 101407, China*

[c]*General Research Institute for Nonferrous Metals, Beijing, 100088, China*

1 Measurement of gas sensing performance

A simple schematic diagram of the JF02 gas sensing system was showed in **Figure S1**. All gases used to test was injected by external gas cylinders and mixed in the "Mixture Chamber". The mass flow and concentration of gases was controlled by the "Mass Flow" module which was monitored though flowmeter. The temperature controlled in the sensing process was realized though the heat platform in the closed "Test Chamber". The resistance signal changes were captured by a pair of electrode probe in the "Test Chamber", processed by the "Test Module" and transmission in to PC.

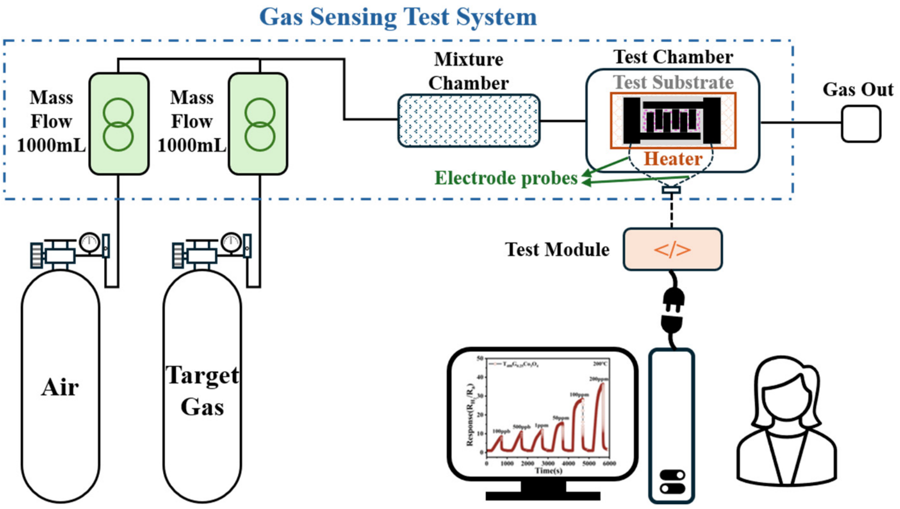

Figure S1. Schematic diagram of JF02 system

2 Morphology and Structure of Porous Urchin-like Co_3O_4

The mass loss of 4 Co_3O_4 precursors are mainly concentrated at 200-300°C, which was calculated to be 19.40%, 21.40%, 20.64% and 20.80% by TGA results respectively. And the material tends to be stable at 400-900°C (**Figure S2**). Calcination temperature can be set to 400°C to ensure that the $Co_2(OH)_2CO_3\cdot11H_2O$ is oxidized to Co_3O_4 and protect urchin-like structure from collapse.

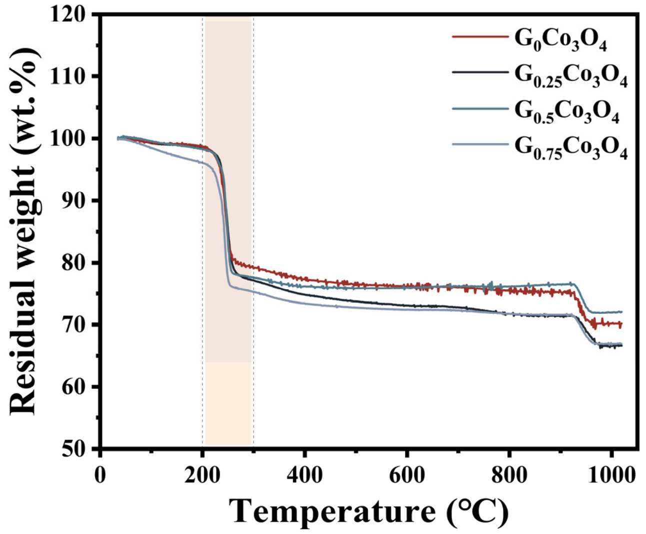

Figure S2. TGA curves of Co_3O_4 precursors

Due to the low crystallinity of the precursor, the XRD pattern showed a large bulging peak (**Figure S3**), and the diffraction peak under the bulging peak corresponds to the $Co_2(OH)_2CO_3\cdot11H_2O$ phase (PDF#48-0083).

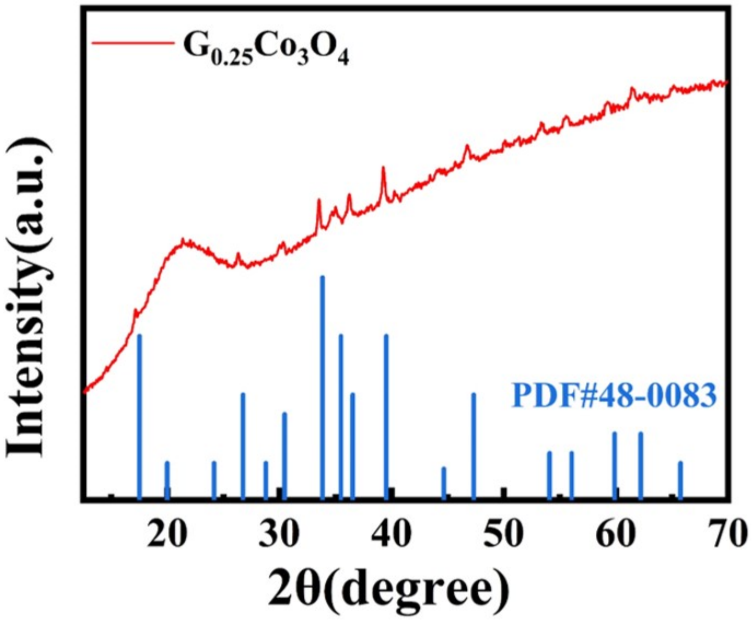

Figure S3. XRD pattern of $G_{0.25}Co_3O_4$

After calcinating process, rod-shaped $G_0Co_3O_4$ is seriously aggregated into bolck, and which is tightly composed of nanoparticles with few stacked pores (**Figure S4a**). Partial nanoneedle structure was retained of $T_{400}G_{0.5}Co_3O_4$ (**Figure S4b**), while $T_{400}G_{0.75}Co_3O_4$ illustrated a structure of near-solid microspheres (**Figure S4c**). The structure above went against to obtain high specific surface area.

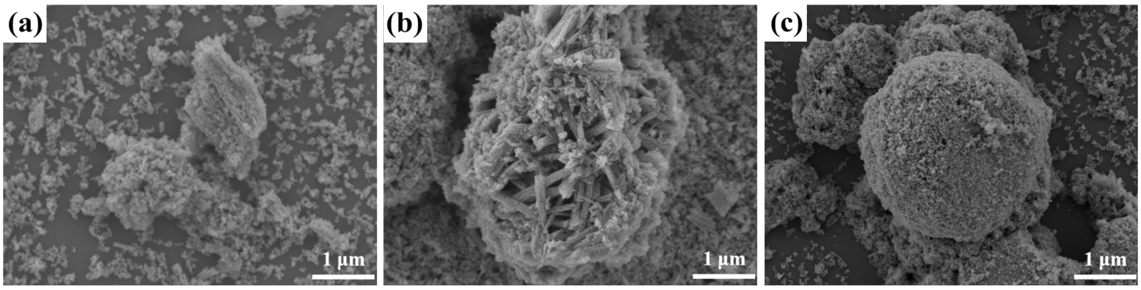

Figure S4. SEM images of $T_{400}G_xCo_3O_4$: (a) $T_{400}G_0Co_3O_4$; (b) $T_{400}G_{0.5}Co_3O_4$; (c) $T_{400}G_{0.75}Co_3O_4$

3 Hydrogen sensing performance of porous urchin-like Co_3O_4

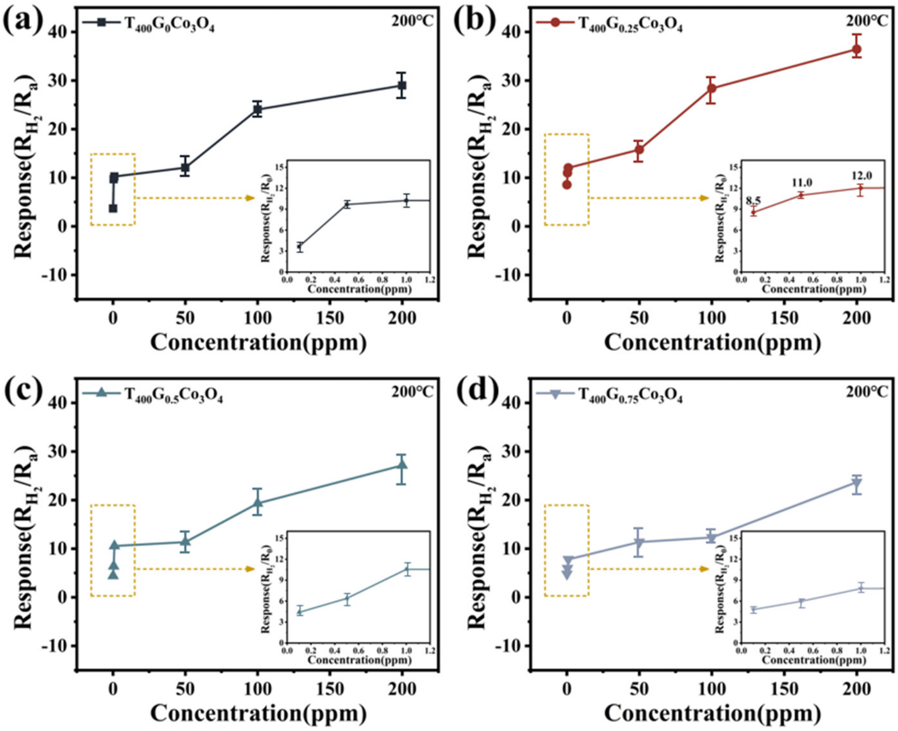

Figure S5. Response to different concentrations of H_2 at 200°C: (a) $T_{400}G_0Co_3O_4$; (b) $T_{400}G_{0.25}Co_3O_4$; (c) $T_{400}G_{0.5}Co_3O_4$; (d) $T_{400}G_{0.75}Co_3O_4$

The response of the material to hydrogen in each cycle is comparable and can return to the initial base value (**Figure S6**), indicating that the material has good repeatability.

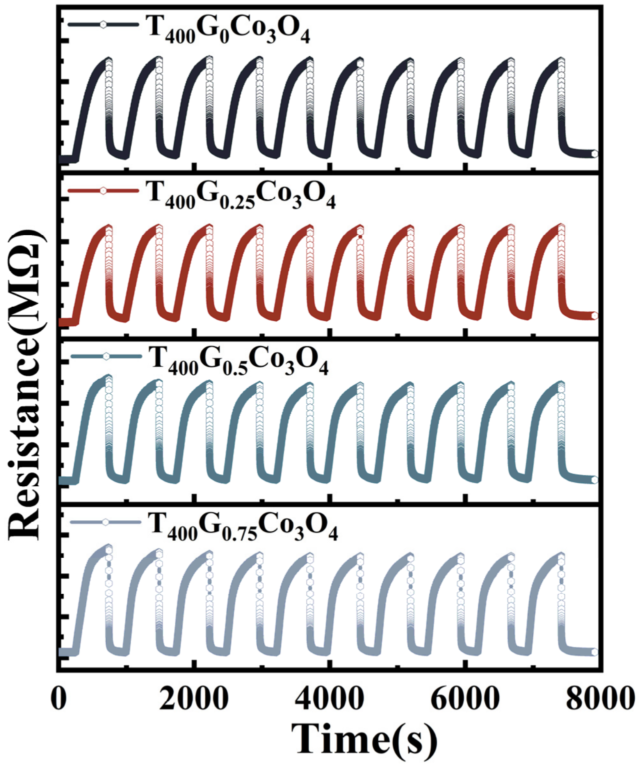

Figure S6. Dynamic response curve of $T_{400}G_xCo_3O_4$ at 200°C after 9 hydrogen cycles
